# On the role of training in delay of gratification paradigms: a reply to Pepperberg 2022

**DOI:** 10.1007/s10071-023-01752-7

**Published:** 2023-02-14

**Authors:** Désirée Brucks, Matthew B. Petelle, Auguste von Bayern, Anastasia Krasheninnikova

**Affiliations:** 1 Max-Planck-Institute for Ornithology, Eberhard-Gwinner-Street, 82319 Seewiesen, Germany; 2 Max-Planck Comparative Cognition Research Station, Loro Parque Fundación, 38400 Av. Loro Parque Puerto de la Cruz, Tenerife, Spain; 3 Animal Husbandry, Behaviour and Welfare Group, Institute of Animal Breeding and Genetics, University of Giessen, Leihgesterner Weg 52, 35392 Giessen, Germany; 4 Center for Proper Housing: Poultry and Rabbits (ZTHZ), Division of Animal Welfare, VPH Institute, University of Bern, Burgerweg 22, Zollikofen, Switzerland

**Keywords:** Delay of gratification, Self-control, Parrots, Comparative cognition experimental design

In our manuscript (Brucks et al. 2021), we investigated delay of gratification across several parrot species using a rotating disc task. Our study highlighted potential issues with previous paradigms while offering a promising novel method for conducting comparative studies in the future. We would like to thank Dr. Pepperberg for the thoughtful commentary on our manuscript (Pepperberg 2022), and we welcome her comments as part of the much needed, constructive dialogue, which will move the field forward.

Pepperberg’s main critique focuses on (1) our characterisation of the “wait” signal used in her study on delayed gratification in a grey parrot (Koepke et al. 2015) and on (2) the training method used in our own study.

## “Wait” signal: a trained command or a learned concept of waiting?

Pepperberg (2022) disagrees with our and others’ (e.g., Miller et al. 2019a; Schwing et al. 2017) interpretation of the “wait” signal used for the African grey parrot “Griffin” in Koepke et al. (2015) as a *trained command*. The author highlights that Griffin was never trained to wait. According to the author, Griffin had gained an *understanding* of the “concept of waiting” in various situations where he had to wait, for example when “his cooked grains cooled on an inaccessible table until they could be eaten” (Koepke et al. 2015). When we refer to the wait signal in Koepke et al. (2015) as a *command*, this does not exclude that he was given a *choice* to wait and could quit waiting at any time. Pairing a cue with an event that requires waiting for food or access to other situations that are highly motivated (e.g., interactions with humans) will lead to an association between the two events eventually. Whether and at what point in time such an association develops from a mere associative one (e.g., “wait” = suppress moving towards enticing reward, as in a command) into a formed concept of waiting (e.g., “wait” = suppress following a desire based on the knowledge that a more advantageous alternative is possible) cannot be determined unless documented and investigated systematically. But irrespective of whether we refer to it as a *trained command* or a *learned concept* of waiting, it remains a matter of fact that Griffin got verbal instructions, which he had previously learned and which he responded to in novel situations. This use of a previously learned (verbal) instruction to “wait” renders the experimental approach different from all other delay of gratification tasks performed with non-human animals. Assuming a species to be tested is capable of generalising such a (verbal) instruction to “wait” to new (experimental) situations reliably, this approach poses the advantage that no or just minimal training is required before the subject can be tested the first time in a delayed gratification paradigm. Additionally, it may be possible to test the subject with varying delays from the start rather than incrementally prolonging the delay stages as in most delayed gratification paradigms to date. However, such an approach is only possible with extensively (language-) trained individuals; thus, excluding the majority of species that could be tested in cognitive studies. Furthermore, it is extremely difficult to ensure that the subjects have acquired the “instruction to wait” reliably and are indeed capable of transferring it to novel situations. Standardising the training procedure for a concept of waiting is impossible as the exposure to the signal is difficult to record. Consequently, replicating the setup with other language-trained individuals is not possible, thus further limiting the applicability for comparative studies.

Delay of gratification paradigms for non-human animals needs to convey the task’s contingencies to the test subjects first as they typically cannot rely and refer to a previously learned wait concept. This requires training, such as demonstration trials with forced choices and successively increasing delay stages across sessions; however, as Pepperberg (2022) correctly noted, the training procedure might interfere with the performance in the test (see below for further discussion). Nonetheless, these delays of gratification paradigms allow us to assess the upper limits of self-control on a comparative level as long as training and testing are well standardised (see Table 1). Given these procedural differences and the differential training or instructions conveyed to the animals between Koepke et al. (2015) and other delay of gratification studies in non-human animals, including our study, the results are not directly comparable (see Table 1). Nonetheless, Griffin’s ability to delay gratification in an experimental paradigm that most likely is more cognitively demanding than other commonly used tasks, is remarkable in itself and we did not intend to denounce Koepke et al.’s (2015) results but rather wanted to emphasise the inherent difference in underlying experimental procedures.

**Table 1:** Procedural differences between delay of gratification studies utilising different experimental paradigms

	Koepke et al. (2015)	Rotating tray task (e.g., Brucks et al. 2021; Miller et al. 2019b)	Exchange task (e.g., Auersperg et al. 2013; Schwing et al. 2017)	Accumulation task(e.g., Hillemann et al. 2014; Vick et al. 2010)
Pre-training procedure				
Food types	7 Types: high (2), medium (3), low quality (2)	2 Types: high, low quality	3 Types: high, medium, low quality	4 Types*: high, medium (2), low quality
Food preference	Four trials each day before test trials	Sessions at beginning of study	Sessions at beginning of study	Sessions at beginning of study
Pilot trials	17 additional trials to examine variation of protocol	None	None	None
Training				
Forced training	Exposure to “wait” instruction within daily routine (procedure not documented)	Habituation to apparatus (i.e., food items placed on first and second position separately)	Exchange training (non-edible item exchanged for edible item)	Demonstration of accumulation out of reach
		Demonstration trials with delay of upcoming test trials between options		Demonstration of accumulation at beginning and end of each session
Free choice exposure	Demonstration of alternative choice when waiting for “ < 5 s” (number of repetitions not specified)			Waiting until all items had accumulated within reach
Training criterion	No criterion	Take food items from rotating tray	Correct execution of exchange behaviour	Successful waiting until accumulation stops
Test				
Experimenter’s behaviour	(1) Showing reward types, baiting cups	(1) Showing reward types, baiting food holders	(1) Showing reward types	(1) Showing reward types
	(2) Hand on LQR saying “wait” + gesture	(2) Starting engine, passive behaviour/leaving test enclosure	(2) Handing LQR to bird while holding HQR in other hand out of reach	(2) Transferring each reward item separately into reach
	(3) Backing up and standing still			
Presentation of reward types	LQR: cup within reach HQR: in cup held by experimenter “several feet away”	LQR: rotates within reach after 5 s HQR: visible, rotating within reach after delay	LQR: handed to beak at beginning of trial HQR: visible, open hand out of reach (approx. 5 cm from beak)	Items transferred manually from visible, out of reach lid to accessible lid
Delays per session	Different delays within same session	One delay stage per session	One delay stage per session	One delay stage per session
Increase in delays	Random selection of delays per trial	Successive increase in delays based on individual success	Successive increase in delays based on individual success	Successive increase in delays based on individual success
Control trials	HQR first	(1) HQR first	HQR first	None
		(2) both LQR)		
Trials per session	4 Trials per day with 15 min break in-between trials	10 Trials per session; < 3 sessions per day	9–20 Trials per session	5–10 Trials per session; < 3 sessions per day (one daily trial for higher delays in Vick et al.)
Criterion for success	None	Wait for HQR in subset of test trials	Wait for HQR in subset of test trials	Wait for 2nd reward in subset of test trials

*HQR* high-quality reward, *LQR* low-quality reward.

*Only one food type of medium quality was used in Vick et al. (2010)

Pepperberg’s critique of our study (Pepperberg 2022) contains four aspects. First, she argues that the parrots in our study were repeatedly subjected to enforced training regimes (i.e., during demonstration trials and in case of decisions for the low-quality reward). Second, she suggests that the parrots were exposed to successive training throughout the course of the test as delay stages increased in incremental steps. Third, Pepperberg comments on the use of associative cues that might have primed the parrots to wait. These cues involve the use of differently coloured food holders, the presence of the low-quality reward, and the fact that the birds received sessions of several trials rather than individual trials randomly presented throughout the day. All these cues might have helped the bird to predict what is going to happen and base their behaviour (i.e., decision to wait) on these cues. And fourth, Pepperberg raises a methodological issue as the food holder was positioned in a way that it was moving away from the birds during longer delay stages.

### (1) Enforced training during demonstration trials and the test

Pepperberg (2022) raises an important point in her comment related to the aspect of training in delay of gratification paradigms. The animals need to know the task’s contingencies to be able to show self-control. Consequently, all studies that use a delay of gratification paradigm also implement a training phase in which the subjects are familiarised with the task and the concept of gaining access to a better food reward only if they refrain from consuming the inferior reward. These initial training steps are usually conducted with a low delay of several seconds to facilitate learning. Only subsequently are the animals presented with higher delays, often in consecutive, incremental steps. Obviously, these procedural steps give the animals ample learning opportunities with the task that might affect their self-control performance to some extent. However, without knowing the task contingencies, it is impossible for any species under examination to show self-control and the study would result in false negatives. For collecting (directly) comparative data on multiple species, it is necessary to use an experimental design that allows animals with different training histories (which are certainly not comparable to Griffin or other parrots in Pepperberg’s lab) to perform correctly in the task. This basic training with the experimental paradigm included exposing the birds to demonstration trials (enforced trials at the beginning of each test session with the respective delay) in which the birds could only access the high-quality reward after a delay had passed without having the possibility to choose an immediately available low-quality alternative. As correctly pointed out by Pepperberg, these demonstration trials resemble forced training, which is necessary to ensure that the birds’ experienced the task’s contingencies, namely that the food holders would move forward and become available after different durations. Crucially, however, the subjects could never practise their self-control in these trials as the apparatus was only pushed within reach once the delay was over and the high-quality reward available simultaneously with the low-quality option. The birds could not access the low-quality reward during the demonstrated delay; thus, were not required to actively abstain from taking the immediately available food option.

Nonetheless, during the test phase, the parrots may have learned cumulatively with each trial via both positive and negative reinforcement to wait for the high-quality reward. If they went for the low-quality option, they saw the now inaccessible high-quality option moving forward, which they likely perceived as frustrating. If they had gone for the high-quality food, they were positively reinforced for waiting. However, the same is true for any experiment with multiple trials, including Koepke et al. (2015). Even if they “learn” to wait, it shows that they have “*capacity* for self-control”, other species may lack irrespective of how much they are trained. More precisely, as mentioned above, the effect of training will have a limit, i.e., no matter how much experience an animal accumulates, at some point a limit in the duration an animal is prepared to wait will be reached. It was the objective of our study to examine this *capacity* across species.

Furthermore, the presentation of the low-quality reward only after a delay of 5 s is an inherent feature of the rotating tray task that is necessary to ensure that the animals are experiencing the rotational movement of both reward types. If the low-quality reward would be immediately available when the apparatus is pushed within reach of the birds, the attention could be drawn towards the moving high-quality reward, which in turn would be even more interesting compared to the stationary low-quality reward. Pepperberg asserts that our training regime with the rotating disc may help improve performance by priming individuals to wait in subsequent trials, but alternatively, seeing food rewards move without being able to access them might also be perceived as frustrating, which could, in turn, lower performance.

### (2) Training with incremental delay steps

Apart from the enforced training during demonstration trials, Pepperberg (2022) also raises the possibility for learning to occur during the test as delay times are increased in a stepwise manner depending on individual success. It is true that this method is likely to achieve longer waiting times than other methods in which delay time is completely random (as, e.g., in Koepke et al. 2015), because waiting for short durations should be less demanding than long waiting times. The task is then made increasingly more demanding but with each step positively reinforced. Yet, if it was indeed happening in the parrots, we would expect to have found an increase in the parrots’ success as the number of trials increased, potentially in particular during long delay times when the birds already had extensive exposure to the task. However, we found no main effect of trial on success and we also see trial success decrease as delay time increases as one would expect given that there should be limits to the parrots’ self-control capacity. Accordingly, in the parrot species, we tested repeated experience with the delay of gratification task likely does not create sufficient training in itself to facilitate performance but rather still allows for individual variation in self-control abilities to occur. If the birds had learned that waiting results in a better reward throughout sessions, we would have expected to see an increase in performance across trials and sessions. Instead, we observed temporal discounting of the high-quality reward in our parrots, resulting in lower performance across trials. Nonetheless, as already said above, we agree with Pepperberg, having repeated exposure to the same problem (i.e., get better reward after delay) certainly affects individual performance in subsequent trials via associative learning. This is a concern that may be evident in most studies of cognition (Heyes 2012). Many animal cognition studies are characterised by a trade-off between ensuring the subjects correctly perform according to the task’s contingencies and keeping the training to a minimum to be able to observe a behavioural reaction that most closely resembles natural individual variation in cognition. Nonetheless, even if trials with randomly varying delay times are presented, as proposed by Pepperberg (2022), learning is likely to occur across repetitions. While we do not know yet to which extent training can affect performance in delay of gratification tests, we know that each individual ultimately reaches his/her indifference point. At this indifference point, the delayed option is devalued to a lower level compared to the immediate option and, thus, waiting no longer pays off (e.g., see Vanderveldt et al. 2016). Even extensive training cannot shift this discounting function to an infinite point as there are biological limits to this capacity. In fact, a study with human participants found that forced training and repeated trials did not affect performance compared to the “human version” of the delay of gratification test with verbal instructions and few test trials (Lagorio and Madden 2005). Furthermore, it could be argued that massing trials, as criticised by Pepperberg (2022), allows to gain more robust data on individual indifference points instead of relying on few trials that could be more easily affected by confounding factors.

### (3) Performance based on associative cues

Pepperberg (2022) further raises concerns with the fact that the birds in our study could use associative learning based on cues provided during the task that might have facilitated their performance. These cues involved the presence of the low-quality reward (since the low/high reward never varied) and the differently coloured food holders. While certainly in the context of the delay of gratification tasks, the presence of the low-quality reward acts as a cue to signal that a better reward will be accessible at some point, we did test whether the birds relied on position learning during the test. In fact, we included a position control trial, during which the high-quality reward was available immediately and the low-quality reward followed after a delay. We found that the birds did not wait for the second option if the high-quality reward was offered first. Accordingly, the birds did not learn the rule of selecting the second option but rather based their behaviour on reward quality. Furthermore, in the low-quality control in which both options included low-quality rewards, we found that the birds could differentiate between the two options and did not wait for the second option if they did not differ in terms of quality. Whether associative cues facilitated the birds’ success in these control trials (i.e., absence of colour associated with high-quality reward) is difficult to assess retrospectively; however, if the birds would have relied on the absence of the colour cue to guide their choices in these control trials, we would have expected to see a more constant performance. Instead, the birds’ success in the low-quality control trials increased across sessions. Contrary to Pepperberg’s assumption that this behaviour shows the control trials provided further training, we argue that success in the low-quality control demonstrates that the birds behaved in line with the task’s contingencies. Having access only to low-quality rewards is a violation of expectation for the birds; accordingly, with increasing exposure to these incongruent control trials (which were randomly interspersed with normal test trials), the birds got more familiar with the peculiarities of the control conditions. Future studies should implement an additional control condition in which both options are high-quality rewards (one available immediately and the other one after a delay) to control for the possibility that absence or presence of colour cues may facilitate subjects’ performance in a rotating tray task.

### (4) Methodological concerns with rotating tray during higher delay times

Pepperberg (2022) criticises that during higher delay times, the high-quality reward was positioned in a starting position with an angle of more than 180° to the bird and, consequently, was first moving away from the bird. We failed to describe in the methods section of our manuscript that the starting position for > 30 s was 180° to the bird. Once the trial started, the arm would move towards the bird, but would halt at the 15 s position until the desired time had elapsed.

## Suggestions for future studies

Several points that were raised by Pepperberg (2022) warrant a broader discussion and should be implemented in future delay of gratification paradigms. The role of training in self-control studies has received only little scientific attention. To our knowledge, no study has systematically varied the degree of training prior to assessing self-control to find out whether training facilitates self-control performance in the subsequent test. For example, animals could be assigned into minimal and maximal training groups or animals that are already familiar with the task could be re-tested after some time has passed without receiving any refreshing training. Furthermore, considering that animals gain access to the same food rewards during training, satiation and subsequent devaluation of rewards during the test need to be considered. While statistically controlling for learning across trials or sessions or recording daily body weights (in case of small species) offers an indirect solution to this issue, future studies need to investigate the influence of satiation with either low-quality or high-quality rewards on subsequent performance in the test.

Another way of circumventing the training issues might be the presentation of delays in a randomised manner—as performed in Koepke et al.’s (2015) study and also in most delay of gratification studies with children. Instead of systematically increasing the delay depending on the individual’s success, the presentation of random delay times can reduce the number of sessions needed to test an individual (and thus reduces the training experience with the task); however, such a procedure also increases the risk of frustrating the animals as the task gets more unpredictable. Furthermore, as suggested by Pepperberg, presenting trials in an interspersed manner (e.g., single trials throughout the day) instead of testing multiple trials in a session certainly provides another interesting solution to reduce the impact of repeated exposure of the task on the animals’ behaviour. If, however, such methodological adjustments are implemented, comparability to other studies and species might be hindered. Nonetheless, it needs to be acknowledged that even with minimal training and few repeated exposures, learning is taking place as this aspect can only be excluded in one-shot setups, which would not prove reliable in the case of establishing indifference points.

Alternatively, it might be interesting to develop novel experimental designs that resemble naturally occurring foraging decisions and thus require no formal training prior to testing the animals. For instance, parrots could be tested with differentially ripened fruits in various locations to mimic a naturally occurring foraging problem.

Another issue with comparative delay of gratification paradigms, that remains unsolved and needs further consideration by future studies, is the derivation of a quantitative measure for reward preferences. Influences of life experiences (i.e., socio-ecological factors and individual experiences, such as hunger) affect the value that is attributed to rewards. While individual-specific experiences can be minimised by testing a large number of individuals with a standardised and known history (e.g., hand-raised animals in captivity), species-specific predispositions are much harder to consider. Nonetheless, even if species do not attribute the same value to the rewards that are being offered, ensuring that the animals are tested with an intuitive setup (with minimal pre-training exposure), pass a standardised training procedure, and are performing in line with the task’s contingencies during controls, is our best shot for assessing self-control on a comparative scale at the moment.
